# Evaluation of in vivo staging of amyloid deposition in cognitively unimpaired elderly aged 78–94

**DOI:** 10.1038/s41380-022-01685-6

**Published:** 2022-07-20

**Authors:** Malgorzata M. Michalowska, Karl Herholz, Rainer Hinz, Chinenye Amadi, Lynn McInnes, Jose M. Anton-Rodriguez, Thomas K. Karikari, Kaj Blennow, Henrik Zetterberg, Nicholas J. Ashton, Neil Pendleton, Stephen F. Carter

**Affiliations:** 1 Wolfson Molecular Imaging Centre, University of Manchester, Manchester, UK; 2 Department of Neuropsychology and Psychopharmacology, Maastricht University, Maastricht, The Netherlands; 3 Division of Clinical Geriatrics, Center for Alzheimer Research, Department of Neurobiology, Care Sciences and Society, Karolinska Institutet, Stockholm, Sweden; 4 Division of Neuroscience and Experimental Psychology, University of Manchester, Manchester, UK; 5 Sheffield Institute for Translational Neuroscience, University of Sheffield, Sheffield, UK; 6 Department of Psychology, Faculty of Health and Life Sciences, Northumbria University, Newcastle upon Tyne, UK; 7 Christie Medical Physics and Engineering, The Christie NHS Foundation Trust, Manchester, UK; 8 Department of Psychiatry and Neurochemistry, Institute of Neuroscience and Physiology, The Sahlgrenska Academy at the University of Gothenburg, Mölndal, Sweden; 9 Department of Psychiatry, University of Pittsburgh, Pittsburgh, PA USA; 10 Clinical Neurochemistry Laboratory, Sahlgrenska University Hospital, Mölndal, Sweden; 11 Department of Neurodegenerative Disease, UCL Institute of Neurology, Queen Square, London, UK; 12 UK Dementia Research Institute at UCL, London, UK; 13 Hong Kong Center for Neurodegenerative Diseases, Clear Water Bay, Hong Kong, China; 14 King’s College London, Institute of Psychiatry, Psychology and Neuroscience Maurice Wohl Institute Clinical Neuroscience Institute, London, UK; 15 NIHR Biomedical Research Centre for Mental Health and Biomedical Research Unit for Dementia at South London and Maudsley NHS Foundation, London, UK; 16 Centre for Age-Related Medicine, Stavanger University Hospital, Stavanger, Norway; 17 Department of Psychiatry, University of Cambridge, Cambridge, UK

**Keywords:** Neuroscience, Predictive markers

## Abstract

Amyloid-beta (Aβ) deposition is common in cognitively unimpaired (CU) elderly >85 years. This study investigated amyloid distribution and evaluated three published in vivo amyloid-PET staging schemes from a cognitively unimpaired (CU) cohort aged 84.9 ± 4.3 years (*n* = 75). SUV-based principal component analysis (PCA) was applied to ^18^F-flutemetamol PET data to determine an unbiased regional covariance pattern of tracer uptake across grey matter regions. PET staging schemes were applied to the data and compared to the PCA output. Concentration of p-tau181 was measured in blood plasma. The PCA revealed three distinct components accounting for 91.2% of total SUV variance. PC1 driven by the large common variance of uptake in neocortical and striatal regions was significantly positively correlated with global SUVRs, APOE4 status and p-tau181 concentration. PC2 represented mainly non-specific uptake in typical amyloid-PET reference regions, and PC3 the occipital lobe. Application of the staging schemes demonstrated that the majority of the CU cohort (up to 93%) were classified as having pathological amount and distribution of Aβ. Good correspondence existed between binary (+/−) classification and later amyloid stages, however, substantial differences existed between schemes for low stages with 8–17% of individuals being unstageable, i.e., not following the sequential progression of Aβ deposition. In spite of the difference in staging outcomes there was broad spatial overlap between earlier stages and PC1, most prominently in default mode network regions. This study critically evaluated the utility of in vivo amyloid staging from a single PET scan in CU elderly and found that early amyloid stages could not be consistently classified. The majority of the cohort had pathological Aβ, thus, it remains an open topic what constitutes abnormal brain Aβ in the oldest-old and what is the best method to determine that.

## Introduction

Deposition of amyloid plaques is an early event in Alzheimer’s disease (AD) pathogenesis [1]. Deposition of amyloid-beta (Aβ) is also observed in up to 38% of cognitively unimpaired (CU) older individuals at age 85 [2] and up to 76% in those carrying at least one copy of the ε4 allele of the apolipoprotein E gene (APOE4). However, it is unclear whether this represents an early stage of AD ultimately leading to dementia, or whether it represents a benign age-associated condition.

Positron emission tomography (PET) imaging allows in vivo visualisation and quantification of brain Aβ [3, 4]. Staging schemes based on post mortem histology and amyloid imaging research across the AD spectrum, including individuals with preclinical AD, suggest a downward spreading pattern starting in the neocortex and progressing toward the striatum [5, 6]. Thal et al. estimated neuropathological Aβ-phases [5] by using thresholds based on standardised uptake value ratios (SUVRs) for neocortex and caudate nucleus [7]. Grothe et al. described [8] and longitudinally validated [9] four stages of regional amyloid deposition on ^18^F-florbetapir PET scans in CU individuals, with partial replication in patients with subjective memory impairment [10]. Mattsson et al. defined three stages based on regional longitudinal cortical amyloid progression rates informed by CSF biomarkers [11] (an extended discussion of Thal’s, Grothe’s and Mattsson’s staging schemes can be found in the Supplementary Information). While there is evidence of good correspondence between PET-based and neuropathological staging in patients with MCI or dementia, discrepant findings were observed in CU controls for low and moderate severity amyloid pathology [12].

Neuropathological studies in CU aged above 80 years [13] or centenarians [14] found little if any relation of cross-sectional cognitive function or previous decline with Aβ staging. Thus, the concept of describing the progression of AD by amyloid staging does not seem applicable in these individuals, who may exhibit resilience against any detrimental effects of amyloid. The regional distribution of amyloid deposits in these people may not be aligned with the regional progression pattern rendering them unstageable. As amyloid-PET is providing quantitative values of amyloid tracer uptake, multivariate techniques such as principal component analysis (PCA) can be used to describe distribution patterns [15]. This has previously been used to study the effects of ageing on cerebral glucose metabolism [16], to distinguish between dementia types [17], with amyloid-PET for discrimination between AD patients and controls [18] or for generating templates for spatial processing [19].

Surviving participants from the Newcastle and Manchester Ageing Cohort [20] have now reached 80 years of age and above. This unique, well-characterised elderly population is, on average, at least 10 years older than many previously published studies. Cognitively unimpaired volunteers from this cohort were included to investigate regional amyloid distribution and to determine associations with cognitive function and established confounding risk factors for amyloid deposition, including age and APOE4 presence. PCA was used to describe the regional amyloid deposition pattern without a priori hypotheses. Published in vivo amyloid-PET staging schemes [8, 9, 11] were also tested to determine if they translate to this CU age group.

## Methods

### Participants

Data were acquired from 75 CU elderly, who were part of the PreclinAD study from the European Medical Information Framework for AD [21]. Detailed inclusion and exclusion criteria are outlined in the Supplementary Information. This study was approved by the Greater Manchester South Research Ethics Committee (ref: 14/NW/011) and participants provided written informed consent.

### Collected demographic and clinical data

Data on age, years of education, and APOE genotypes were collected [21]. Scores from the Mini-Mental State Examination, Addenbrooke’s Cognitive Examination-Revised (ACE-R), CERAD, Rey Auditory Learning Test (RAVLT), and Rey Complex Figure Delayed Test (RCFT Delayed) were collected for an overview of cognitive performance. Based on the APOE genotypes, participants were dichotomised into individuals carrying at least one copy of the ε4 allele (APOE4+) and APOE4 non-carriers (APOE4−).

### MRI and PET data acquisition

MRI was performed on a 3T Philips Achieva scanner with a 32-channel head coil including a 3D-T1 with sagittal turbo field echo sequence (1.0 mm isotropic voxels, repetition time = 7.9 ms, echo time = 4.5 ms, flip angle = 8 degrees) for image processing. Radiosynthesis of ^18^F-flutemetamol was performed at the Wolfson Molecular Imaging Centre, University of Manchester. All PET data were acquired on a high-resolution research tomograph (Siemens/CTI, Knoxville, Tennessee, USA). Following an intravenous injection of 185.07 ± 10.5 MBq ^18^F-flutemetamol, PET data were acquired from 90–110 min. A 7 min transmission scan using a 137Cs point source was acquired for attenuation and scatter correction [22, 23]. PET images were realigned to correct for inter-frame motion and reconstructed with an implementation of 3D iterative ordinary Poisson ordered subset expectation maximisation with 12 iterations, 16 subsets and resolution modelling [24–27] using 1.22 mm isotropic voxels. A post-reconstruction Gaussian filter of 4 mm FWHM was used to reduce noise [21].

### Blood plasma p-tau181

Blood samples were collected and frozen on the same day as the PET scan. Plasma p-tau181 concentration was measured using in-house Single molecule array (Simoa) methods on Simoa HD-X instruments (Quanterix, Billerica, MA, USA) at the University of Gothenburg. Methods are described in the Supplementary Information and detailed elsewhere [28, 29]. No blood samples were acquired for 1 participant and 1 sample was excluded due to being >3 SD outside the cohort mean, leaving 73 p-tau181 samples for analysis. To dichotomise the blood plasma p-tau into T−/T+ groups, a cut-off of 17.7 pg/ml was used [30].

### Data processing

Using SPM12 (Statistical Parametric Mapping, Wellcome Trust Centre for Neuroimaging, UCL, UK) with MATLAB R2019b (The MathWorks, Inc., Natick, MA, USA) PET images were coregistered and resliced to the T1-weighted image by rigid body transformation. The T1 images were then segmented into grey matter (GM), white matter (WM), and cerebrospinal fluid (CSF). The Hammers n30r85 probabilistic atlas [31] was inversely warped into native T1 space. GM binary images were created by thresholding the segmented GM images at 0.5. A GM atlas was created by multiplying the GM binary image with the inversely warped Hammers atlas and then used to sample the coregistered PET images, generating mean kBq/ml for 85 brain regions. Harvard-Oxford and Desikan-Killiany (freesurfer) [32] atlases were inverse warped into native T1 space and restricted to GM voxels for each participant so that the percentage of suprathreshold amyloid within the stages established by Grothe [8] and Mattsson [11] could be determined.

For improved spatial accuracy of basal ganglia region definition, Diffeomorphic Anatomical Registration Through Exponentiated Algebra (DARTEL) was added [33]. The standard DARTEL pipeline from SPM was used with segmented GM, WM, and CSF images to transform PET images and the Hammers atlas into the study-specific DARTEL template space, where putamen and caudate nucleus were manually delineated in the axial plane.

To avoid overrepresentation of smaller regions, the orbitofrontal cortex regions (straight gyrus, anterior orbital gyrus, medial orbital gyrus, lateral orbital gyrus, posterior orbital gyrus) were merged, as well as small basal ganglia regions (nucleus accumbens, substantia nigra, pallidum). Thus, standardised uptake values (SUVs) of 67 GM regions entered into the PCA.

A non-standard centiloid (CL) pipeline was adopted following the directions specified in Klunk et al. [34] with Hammers’ atlas cerebellar GM as reference region as outlined in the Supplementary Information (Supplementary Figs. 1–3). Amyloid positivity (Aβ+) was determined globally and regionally with published cut-offs established with in vivo [35] and post mortem data [36]. Global positivity was defined as >29 CL (>1.48 SUVR) and within stage regions [8, 11] >12 CL (>1.31 SUVR) was considered positive. Between 12–29 CL is classed as the “grey zone”. For Aβ phase estimates from Thal [7], the specific, published cut-offs, with pons as reference region, were used.

### Statistical analyses

Statistical analyses were performed with SPSS 25 (SPSS Chicago, IL, USA) using a *p* < 0.05 threshold of statistical significance. Independent *t*-tests compared demographic and clinical data between groups (with adjustment for unequal group size where appropriate). Multiple regression assessed whether cognitive scores are predicted by age, APOE4, or education years. Amyloid distribution across 67 GM regions was examined by entering SUVs into a PCA, to uncover possible interrelations between brain regions [37]. Regions loading highly on the same principle component (PC) would indicate comparable ^18^F-flutemetamol retention, thus allowing for a regional depiction of amyloid deposition. SUVs instead of SUVRs permitted a reference region-independent representation of covariation of amyloid accumulation. The suitability of the data for PCA was tested with the Kaiser-Meyer-Olkin (KMO) test. The correlation matrix was assessed to ensure that the correlations between the regional SUVs are significant and exceed *r* = 0.30. Components were extracted with a criterion of an eigenvalue above 1. An orthogonal varimax rotation ensured that PCs were uncorrelated with each other, thus improving their interpretability. Finally, each individual’s regression-based component loading was saved for extracted PCs.

The effects of APOE4, age, and years of education on regional amyloid deposition (expressed by individual PC loadings) were examined with Pearson partial correlations. For exploratory purposes, cognitive measures were correlated with the PC loadings, adjusting for APOE4, age, and years of education years. The individual loadings were also correlated with CL/SUVRs. The percentage of suprathreshold amyloid deposition was reported according to the staging schemes discussed above [7, 8, 11].

## Results

### Demographic characteristics and cognition

Table 1 summarises the cohort. Significant differences were found between APOE4− and APOE4+ in terms age and RCFT delayed. Delayed RCFT was significantly different between middle-old and oldest-old individuals (see Supplementary Table 1). Multiple regression predicting RCFT-delayed from APOE4 status and age was significant (*F*_(2, 72)_ = 8.42, *p* = 0.001, *R*^2^ = 0.17) but only age contributed to the model significantly (*B* = −0.53, *p* = 0.001). Total ACE-R scores were significantly correlated with years of education, accounting for age and APOE4 (*r* (70) = 0.30, *p* < 0.05), as well as with CERAD Delayed (*r* (68) = 0.50, *p* < 0.001), RAVLT delayed (*r* (68) = 0.55, *p* < 0.001), and RCFT delayed (*r* (68) = 0.25, *p* < 0.05).

**Table 1:** Characteristics of the cohort.

Measure	Overall	Aβ−	Aβ+	APOE4−	APOE4+
Age (years)	84.9 ± 4.27	85.4 ± 4.08	84.4 ± 4.44	85.5 ± 4.26	82.7 ± 3.67^a^
Sex (F/M)	58/17	25/10	33/7	43/14	15/3
Education (years)	14.2 ± 2.98	15.1 ± 3.17	13.3 ± 2.55	14.3 ± 3.18	13.9 ± 2.26
MMSE (/30)	28.8 ± 1.24	29 ± .93	28.6 ± 1.41	28.8 ± 1.13	28.7 ± 1.53
ACE-R total (/100)	92.68 ± 4.91	93.11 ± 4.35	92.3 ± 5.39	92.6 ± 4.86	92.9 ± 5.21
ACE-R Memory (/26)	23.2 ± 2.83	23.1 ± 2.71	23.3 ± 2.95	23.2 ± 2.97	23.2 ± 2.39
CERAD Delayed (/10)	8.15 ± 1.54	7.83 ± 1.63	8.43 ± 1.41	8.18 ± 1.53	8.06 ± 1.58
RAVLT Delayed (/15)	10.6 ± 3.32	10.2 ± 3.39	11.0 ± 3.25	10.7 ± 3.21	10.4 ± 3.72
RCFT Delayed (/36)	14.9 ± 6.17^b^	15.0 ± 5.24	14.8 ± 6.95	14.0 ± 5.78	17.6 ± 6.7^a^
p-tau181 (pg/ml; *n* = 73)	16.3 ± 5.64	14.7 ± 5.14	17.7 ± 5.72^c^	15.8 ± 5.82	17.9 ± 4.79
	***n*** **(%)**	***n*** **(%)**	***n*** **(%)**	***n*** **(%)**	***n*** **(%)**
Aβ+ (>29 CL\1.48 SUVR)	40 (53.3)	35 (46.7)	40 (53.3)	26 (45.6)	14 (77.8)
APOE4−	57 (76)	31 (88.6)	26 (65)	57 (100)	–
APOE4+	18 (24.3)	4 (11.4)	14 (35)	–	18 (100)
APOE E2/E4	3 (4.1)	1 (2.9)	2 (5)	–	3 (16.7)
APOE E3/E4	14 (19.2)	3 (8.6)	11 (27.5)	–	14 (77.8)
APOE E4/E4	1 (1.4)	–	1 (2.5)	–	1 (5.6)
Middle-old (78–84 years)	38 (50.7)	15 (42.9)	23 (57.5)	25 (43.9)	13 (72.2)
Oldest-old (≥85 years)	37 (49.3)	20 (57.1)	17 (42.5)	32 (56.1)	5 (27.8)

Values are mean ± standard deviation, or number of participants in a subset of the sample (*n*).

*MMSE* Mini-Mental State Examination, *ACE-R* Addenbrooke’s Cognitive Examination-Revised, *CERAD* Consortium to Establish a Registry for Alzheimer’s Disease, *RAVLT* Rey Auditory Learning Test, *RCFT* Rey Complex Figure Test, *CL* centiloid, *APOE* apolipoprotein E.

^a^Significant difference (*p* < 0.05) between APOE4− and APOE4+ according to the independent samples *t*-test.

^b^Significant difference (*p* < 0.05) between middle- and oldest-old individuals according to the independent samples *t*-test.

^c^Significant difference (*p* < 0.02) between Aβ− and Aβ+ individuals according to the independent samples *t*-test.

### Pattern of amyloid deposition based on principal components analysis

**Fig. 1:**
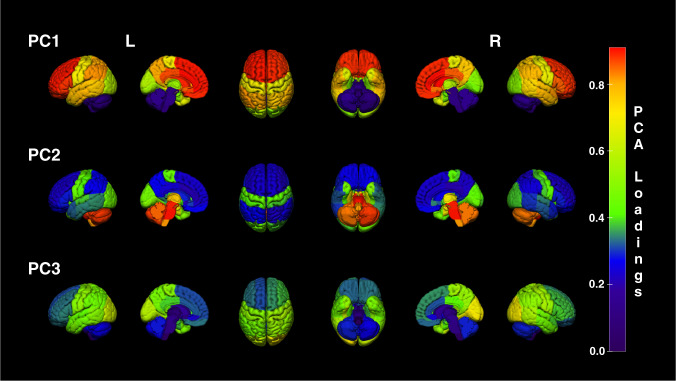
Representation of regional loadings from the first three PCs. Each Hammers atlas region was scaled to its loading score from the PCA. The 3D rendering clearly demonstrates the highest loadings for PC1 were obtained in frontal neocortex, followed by striatum (see Supplementary Fig. 4). Highest loadings for PC2 were found in the brainstem and cerebellum, which are often reference regions for SUVR calculations. Highest loadings for PC3 were found in the occipital cortex. The same data are tabulated in Supplementary Table 2. Colour bar represents PC loadings from highest (red) to lowest (violet). PC principal component, L left, R right.

The KMO test of sampling adequacy yielded a value of 0.83 indicating the data was suitable for PCA. Three PCs were extracted accounting for 91.2% of the total variance. Initial eigenvalues indicated the first two extracted PCs accounted for 88.9% of the total variance. After the varimax rotation, the first two components explained 52.8% and 21.3% of the variance respectively, while PC3 accounted for 17%. PC1 was driven by mean SUVs of neocortical and striatal regions (Fig. 1). The highest loadings >0.85 came from the anterior cingulate, caudate nucleus, orbital frontal cortex, and middle frontal gyrus (Supplementary Table 2). Subcortical regions, including midbrain, medulla, cerebellum, pons, and thalamus, contributed mostly to PC2 (Supplementary Fig. 4). PC3 reflected SUVs of occipital lobe areas. Hippocampus, parahippocampus, and amygdala loaded on all PCs, with highest loadings of 0.7 on PC2.

### Correlation between PCs and global CL\SUVR

**Fig. 2:**
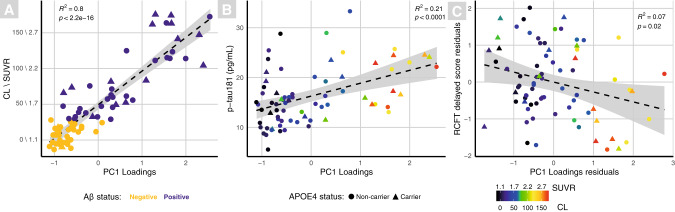
Scatter plots displaying significant correlations with PC1. Each scatter plot shows a significant correlation between global CL\SUVRs and PC1 (**A**), blood plasma p-tau181 and PC1 (**B**), and the significant partial correlation (adjusted for age, years of education and APOE4) between delayed RCFT and PC1 (**C**). Triangles represent APOE4 carriers and circles APOE4 non-carriers. Binary Aβ status is depicted in **A** (Aβ− = yellow; Aβ+ = violet), **B**, **C** are coloured by continuous CL\SUVRs, represented by the colour bar.

A highly significant positive Pearson’s correlation was found between with PC1 and global CLs (*r* = 0.90, *p* = <0.0001), Fig. 2A; a significant negative correlation with PC2 (*r* = −0.27, *p* < 0.05); and a significant positive correlation with PC3 (*r* = 0.23, *p* < 0.05; Supplementary Fig. 5).

### Effects of APOE4, age, and education on principal components

A significant positive partial correlation was revealed between the presence of APOE4 and PC1 loadings (*r* = 0.29, *p* < 0.05). Years of education were significantly negatively correlated with PC1 after adjusting for age and APOE4 (*r* = −0.26, *p* < 0.05). Age did not significantly correlate with any of the extracted component loadings after controlling for APOE4 and years of education.

### Blood plasma p-tau181

The p-tau181 concentration was significantly higher in the Aβ+ group (Table 1). A significant positive Pearson’s correlation was found between PC1 and p-tau181 (*r* = 0.46, *p* < 0.0001; Fig. 2B). Using Aβ statuses with p-tau181 concentrations 27 individuals (36.9%) were classified as A−T− and 15 (20.5%) were A+T+. There were no differences in age, education or cognition for the AT groups (see Supplementary Table 1 for breakdown).

### Correlation between cognitive measures and principal components

All cognitive tests indicated negative associations with PC1, but only the RCFT delayed score was significantly negatively correlated (*r* = −0.27, *p* < 0.05; Fig. 2C). Delayed CERAD was significantly negatively correlated with PC2 component loadings (*r* = −0.25, *p* < 0.05; Supplementary Fig. 6).

### In vivo staging schemes

**Fig. 3:**
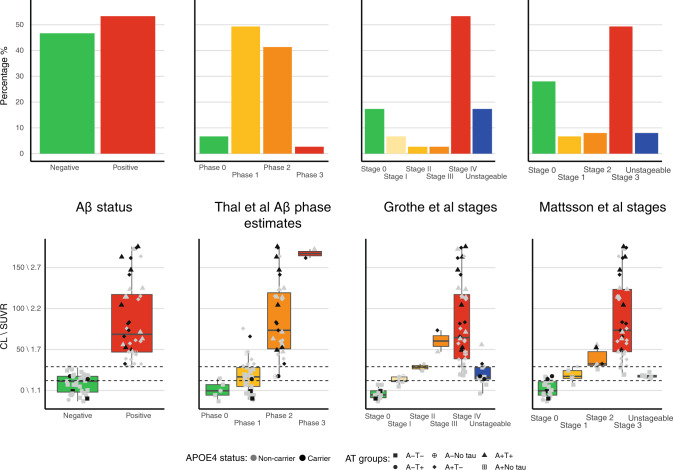
Bar and box plots displaying the percentage of suprathreshold Aβ within brain regions defined by centiloid cortex and 3 in vivo amyloid staging schemes [7, 8, 11]. The top row shows the percentage of individuals categorised within each amyloid phase/stage, while the bottom row displays the global amyloid (CL\SUVR) for those individuals within each phase/stage. In the bottom row, shape of points represents AT status and colour of points represents APOE4 status (grey non-carriers and black carriers); dotted lines in the box plots represent the boundaries of the grey zone (12–29 CL). The figure demonstrates that independent of method, the majority of the cohort is categorised as having pathological quantities of Aβ. Each staging scheme reveals that those individuals at advanced stages tend to also have higher global CL\SUVRs.

Figure 3 depicts good correspondence between Aβ positivity (>29 CL, 53.3% of cohort) and combined high stages of staging schemes (Thal Phase 2 and 3, Grothe Stage III and IV, Mattsson stage 2 and 3). Binary classification into combined high or low stages showed 74 to 91% correspondence between schemes. Both CL and PC1 values provided good discrimination between high and low stages with areas under the receiver operating curves of 0.86 to 0.96. Substantial differences were noted for low stages between staging schemes. Thal’s scheme classified 93.3% of the cohort as PET Aβ Phase estimate ≥1. Only 6.7% were Aβ- by Thal, 17.3% by Grothe and 28% by Mattsson. Grothe’s scheme deemed 17.3% and Mattsson’s 8.0% as unstageable, mostly in the low range of CL values (12–29 CL), which is considered the “grey zone” [35]. There was also little correspondence between CL and PC1 values at these low values (Fig. 2A).

**Fig. 4:**
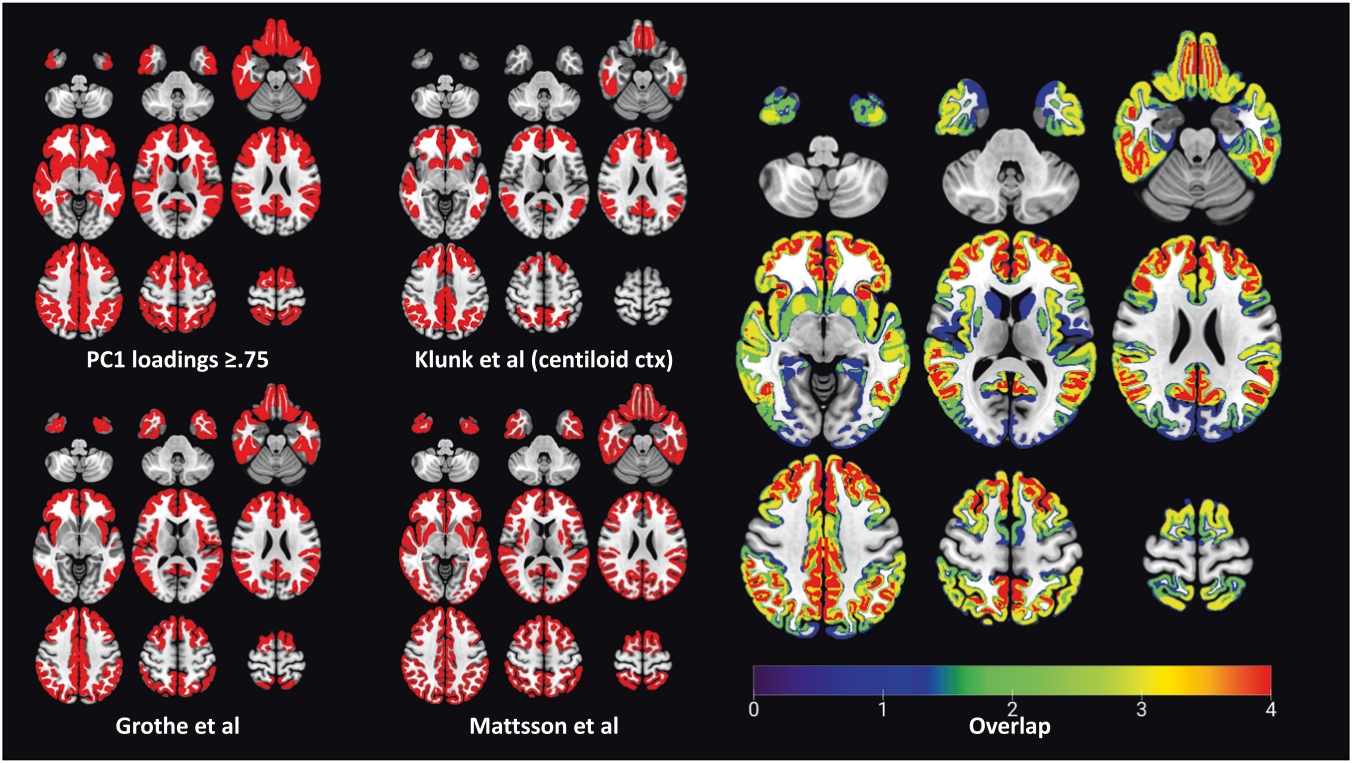
Representation of early and moderate regional amyloid deposition by four different PET-based amyloid deposition schemes. The upper left figure depicts regions showing highest loadings for PC1 (≥0.75), including the frontal, parietal and temporal neocortex, caudate, and putamen. The bottom left figure illustrates stages I and II of the four-stage model of regional amyloid progression proposed by Grothe [8]. The upper middle figure shows cortical volume of interest from the centiloid method established by Klunk [34]. The bottom middle figure depicts the early and intermediate Aβ stages from the longitudinal CSF/PET staging proposed by Mattsson [11]. The brain rendering on the right illustrates the degree of regional overlap between these four methods, which the colour bar depicts; all four methods = red; single method = blue.

Figure 4 depicts the spatial overlap between PC1, centiloid cortex, and the early/intermediate stages from in vivo schemes developed by Grothe [8] and Mattsson [11]. There is a clear regional overlap between all methods, largely within default mode network regions including anterior cingulate, orbital-, middle-, superior-frontal gyrus, posterior cingulate, and insula. Mattsson also classified the putamen as an intermediate amyloid accumulation region, which is relatable to the PCA showing comparable loadings of both neocortex and striatum on PC1. Regions of the centiloid cortical mask [34] also overlapped with PC1, particularly frontal, temporal, posterior cingulate and parietal cortices, but also the anterior striatum and insula. All the extracted PCs were significantly correlated with CLs derived from the centiloid cortex (Fig. 2B and Supplementary Fig. 5A, B).

## Discussion

This study used SUV-based PCA to investigate the regional pattern of amyloid deposition in a unique cohort of CU adults with a mean age of 85 years. It also applied three recently published in vivo Aβ staging schemes to the PET images. The PCA revealed three PCs based on mean ^18^F-flutemetamol SUVs, indicative of differential amyloid burden in these areas. PC1 was driven by the large common variance of uptake in neocortical and striatal regions, PC2 represented non-specific uptake in typical reference regions for SUVR-based analyses, and PC3 represented the occipital lobe. The application of the Aβ staging schemes demonstrated that the majority of the cohort were classified as having pathological amount and distribution of Aβ.

The striatum (caudate/anterior putamen) was included in PC1, Mattsson [11], Klunk [34] and was also a key region in Thal’s staging [7]. While the striatum is not regarded as an early amyloid accumulation region [8, 38], there was a tight covariation between neocortical and striatal regions in the current study, which might be related to the high age of the participants. It has been previously reported that older CU show advanced amyloid pathology including deposition in the striatum [14].

The current analysis did not use a reference region and was based on SUVs. Reference regions, such as pons and cerebellum, often used as a denominator in SUVRs, appeared within PC2. PC2 had a negative correlation with CL\SUVRs. Observing low variation and independence of PC2 from cortical regions, further supports using them as reference regions. Neocortical regions also had low loadings on PC2.

Occipital cortex showed highest PC3 loadings, indicating independent variation compared to neocortical and striatal areas. This could be explained by low occipital amyloid burden and conformational differences of amyloid deposits in the occipital lobe [39], especially since the occipital areas, including cuneus and lingual gyrus, are classified as late amyloid accumulation areas by Grothe’s cross-sectional [8] and as intermediate by Mattsson’s longitudinal staging schemes [11]. Participants with generally high amyloid load, either had high or low occipital load, which did not correlate with APOE4 status (Supplementary Fig. 7). Thus, a factor other than APOE4 may be governing amyloid accumulation in this region.

The APOE4 effect on increased ^18^F-flutemetamol uptake in PC1 is consistent with previous research. APOE4 has been shown to increase amyloid burden in anterior cingulate, posterior cingulate, prefrontal, parietal and lateral temporal areas [40, 41]. These regions belong to well-established brain networks, including the default mode network, showing connectivity alterations and preferential amyloid accumulation in preclinical AD stages in asymptomatic APOE4+ individuals [42–45].

Although amyloid-cognition correlations are debated, some studies reveal a declining cognitive functioning linked to amyloid deposition, even in CU individuals [40, 46]. A weak, significant, negative partial correlation was found between the delayed RCFT and PC1 loadings (Fig. 2B). Worse RCFT performance, a visuospatial episodic memory test, has been linked to hypometabolism and cortical thinning of parietal, temporal, and frontal cortices [47, 48]. A FDG-PET-based PCA also demonstrated correlations between RCFT and metabolism in posterior cingulate, precuneus, parietal, lateral temporal, superior-frontal and medial prefrontal cortices [49]; all these regions loaded highly on PC1. Another weak but significant partial correlation was observed between CERAD delayed and PC2 loadings. Decreased GM density in regions with moderate loadings on PC2, such as the hippocampus, parahippocampal gyrus and thalamus, has been previously associated with worse CERAD immediate and delayed recall [50]. However, we did not find significant correlations between SUVRs of those regions and CERAD delayed.

Participants of this study were also categorised according to their Aβ deposition stage based on three schemes [7, 8, 11]. On a global level, 53.3% of the cohort were Aβ+ (CL >29) and 21.3% were in the “grey zone”. Age-related factors need to be considered when assessing pathological deposits [51] because in non-demented elderly, older age can shift the amyloid distribution to higher values [52]. When applying Thal’s methodology, which uses cortical and caudate SUVRs to stage individuals, 97.3% were at Aβ phase estimate 1 or above, with 44% in phase 2 or above. These phases would correspond to pathological quantities of Aβ post mortem. Although derived differently (frequency of Aβ positivity vs. longitudinal increases of Aβ), both Grothe and Mattsson’s schemes had similar patterns with most individuals categorised in the latest stages (Stage IV and Stage 3; 53.3% and 49.3% respectively), with few individuals in the intermediate stages. Importantly, if the cut-offs used by Grothe (^18^F-florbetapir SUVR of 0.92 converted to −21 CL using the published conversion [53]) and Mattsson (^18^F-flutemetamol SUVRs of 0.738, 0.76 and 0.751 for Stages 1–3 respectively) were used on the current cohort, 100% would have been categorised in the highest stages (Stage IV and Stage 3).

This CU cohort is unique due to its high mean age of 85 years. The above-discussed staging schemes were created based on individuals who were on average at least 10 years younger. Risk factors for dementia differ in this age group when compared to younger elderly. Imaging biomarkers may also have limited applicability due to lower life expectancy and potential resilience to amyloid deposition [54]. Non-demented individuals at age of 85 years might show highly prevalent WM lesions, as well as global and hippocampal atrophy [55, 56]. Tau pathology also increases with age [57, 58] and although tau-PET was not available for the current cohort, plasma p-tau181 revealed a significant positive correlation between PC1 and p-tau181. Dichotomising amyloid-PET and plasma p-tau181 revealed 20.5% were classified as A+T+, thus having evidence of AD pathology without cognitive impairment. Previous multivariate analyses, including PCA, have been used to investigate the effects of age and amyloid on cognition in CU elderly [59]. Although dichotomous amyloid burden has been studied in the oldest-old, aged 85 years and more [2], there is limited neuroimaging research depicting detailed regional amyloid distribution in this old population. This study is novel because it not only assessed regional amyloid deposition, it also applied published in vivo amyloid staging schemes.

When aducanumab was initially approved for clinical use in the USA [60] the presence and removal of brain Aβ became a pressing and highly topical issue [61–63]. It is still unclear whether pathological brain Aβ is as relevant at advanced ages because many Aβ+ oldest-old will never develop cognitive impairment or dementia in their lifetime. This is important because administering a monoclonal antibody to a person who does not need it, is not only expensive but can actually cause harm (e.g., ARIA-E). The present data indicates the uncertainty whether the hierarchal, regional progression patterns based on the in vivo PET staging can be applied cross-sectionally to oldest-old cohorts, and whether positivity cut-offs established on younger populations apply in the ninth and tenth decades of life. Therefore, an open question remains about what constitutes abnormal Aβ in the oldest-old and whether Aβ should be removed from such people’s brains.

This study has limitations. The cross-sectional nature of the data does not allow for conclusions on causal relationships between the variables. Longitudinal research would be essential for assessing the relationship between increasing age, cognitive decline, and progressive changes in Aβ deposition. Due to the data-driven nature of PCA, the modest sample size, as well as the old age of the participants, it’s unclear whether the extracted PCs would generalise to independent younger or diseased cohorts. Moreover, findings based on ^18^F-flutemetamol PET data are only a proxy for actual Aβ neuropathology. Finally, the PCA was conducted on mean regional SUVs, which are dependent on the accurate placement of the regions of interest but are less confounded by noise than voxel-wise PCA [64].

In conclusion, although PC1 and staging schemes broadly overlapped, there was poor correspondence between schemes with respect to early Aβ stages based on regional thresholds. The PCA demonstrated concurrent accumulation across striatum and most cortical regions (apart from hippocampus and occipital cortex), which contrasts with sequential regional accumulation proposed by staging schemes. The data also indicate that a large proportion (up to 93%) of CU elderly have brain Aβ deposits classified as pathological by in vivo PET staging schemes. The study therefore raises important questions about the utility of staging, as opposed to binarising, amyloid-PET from a single scan in the oldest-old, what constitutes abnormal brain Aβ in this age group, and what, if anything, should be done therapeutically for PET Aβ+ CU individuals 85 years of age and above?

## Supplementary information

Supplementary Information

## Data Availability

Requests from suitably qualified individuals for data supporting the findings of this study will be considered by the corresponding author and subject to a data transfer agreement with the host university (University of Manchester).
