# Supplementary material for: Evaluation of in vivo staging of amyloid deposition in cognitively unimpaired elderly aged 78–94
Source: Mol Psychiatry. 2022 Jul 20;27(10):4335–42. doi: 10.1038/s41380-022-01685-6 (PMC9718666; doi:10.1038/s41380-022-01685-6)
Supplement: Supplementary file 1 — Supplementary Information [file 41380_2022_1685_MOESM1_ESM.docx]

**Supplementary Information**

**Contents**

[Details of the three staging schemes applied 2](#_Toc101530050)

[Regions involved in the Thal’s staging scheme 2](#_Toc101530051)

[Regions involved in the Grothe’s staging scheme 2](#_Toc101530052)

[Regions involved in the Mattsson’s staging scheme 2](#_Toc101530053)

[Comparison of the staging schemes 2](#_Toc101530054)

[Inclusion Criteria for the Cognitively Unimpaired Elderly Cohort 3](#_Toc101530055)

[Exclusion Criteria for the Cognitively Unimpaired Elderly Cohort 3](#_Toc101530056)

[Blood plasma processing 3](#_Toc101530057)

[Centiloid processing 4](#_Toc101530058)

[Standard centiloid pipeline 4](#_Toc101530059)

[Non-standard centiloid pipeline 4](#_Toc101530060)

[Supplementary Fig. 1 Published centiloid values vs locally generated values 5](#_Toc101530061)

[Supplementary Fig. 2 Published centiloid values vs non-standard local values 5](#_Toc101530062)

[Supplementary Fig. 3 ^18^F-flutemetamol to 11C-PIB conversion with non-standard pipeline 6](#_Toc101530063)

[Supplementary Table 1 Cohort characteristics based on middle-, oldest-old and AT classification 7](#_Toc101530064)

[Supplementary Table 2 Regional Mean SUVs and Principal Components Loadings for PCs1-3 8](#_Toc101530065)

[Supplementary Fig. 4 Representation of regional loadings from the first three PCs for subcortical regions not visible in Fig. 1 10](#_Toc101530066)

[Supplementary Fig. 5 Scatter plots displaying the significant correlation between global CL \ SUVRs, PC2 (A) and PC3 (B) 11](#_Toc101530067)

[Supplementary Fig. 6 Scatter plot showing the significant partial correlation (adjusted for age, years of education and APOE4) between delayed CERAD and PC2 11](#_Toc101530068)

[Supplementary Fig. 7 Scatter plot showing the relationship between PC1 and PC3 12](#_Toc101530069)

[References 12](#_Toc101530070)

# Details of the three staging schemes applied

## Regions involved in the Thal’s staging scheme

Thal et al^1^ established PET-Aβ phase estimates to distinguish neuropathological amyloid phases with 18F-flutemetamol PET, with pons being used as the reference region. The PET-Aβ phases have been estimated by applying diverse thresholds for the cortical and caudate nucleus (anterior aspect) SUVRs. The first two neuropathological Aβ phases are not detectable on 18F-flutemetamol PET. Therefore, they are marked as PET-Aβ phase estimate 0. Neuropathological Aβ phase 3 is referred to as PET-Aβ phase estimate 1, where cortical SUVR is equal to or higher than 0.5 or caudate nucleus SUVR is equal to or higher than 0.6. Neuropathological Aβ phase 4 is referred to as PET-Aβ phase estimate 2, where cortical SUVR is equal to or higher than 0.6 or caudate nucleus SUVR is equal to or higher than 0.7. Aβ phase 5 is referred to as PET-Aβ phase estimate 3, where caudate nucleus SUVR is equal to or higher than 1.0.

## Regions involved in the Grothe’s staging scheme

Grothe et al^2^ developed an in vivo staging of amyloid deposition based on cross-sectional 18F-florbetapir PET data. They distinguished between four stages of regional amyloid progression. The first stage includes inferior temporal and fusiform gyri, anterior cingulate gyrus, and the parietal operculum. The second stage includes other temporal, frontal and parietal associative areas. The third stage includes primary sensory-motor regions, such as precentral and postcentral gyri, occipital pole, as well as anterior medial temporal lobe. Lastly, the fourth stage shows that the latest region of amyloid deposition is the striatum.

## Regions involved in the Mattsson’s staging scheme

Mattsson et al^3^ developed a three-stage Aβ pathology scheme based on longitudinal multicenter 18F-florbetapir PET data. Based on their staging, the early Aβ accumulating regions include the precuneus, posterior cingulate, isthmus cingulate, insula, as well as medial and lateral orbitofrontal areas. The intermediate Aβ accumulating regions include banks of the superior temporal sulcus, superior temporal, superior frontal and parietal, rostral and caudal middle frontal, frontal pole, fusiform, inferior parietal, inferior temporal, cuneus, lateral occipital, middle temporal, pars opercularis, pars orbitalis, pars triangularis, parahippocampus, putamen, rostral anterior cingulate, as well as supramarginal areas. The latest Aβ accumulating regions are the lingual, pericalcarine, precentral, paracentral and postcentral areas.

## Comparison of the staging schemes

All three staging schemes estimate amyloid stages based on in vivo amyloid PET data. Grothe and Mattsson both use 18F-florbetapir PET and are derived from ADNI data, Thal’s staging is based on a separate cohort using 18F-flutemetamol PET. Only Mattsson’s staging scheme is based on a longitudinal dataset. Mattsson’s staging is also the only scheme that used cerebrospinal fluid data (Aβ42) to estimate amyloid phases. Every staging scheme applies a different method to establish the amyloid phases. Thal’s four stages are based on the whole cortex and caudate nucleus SUVRs, to which higher SUVR thresholds are applied with every progressing amyloid deposition stage. In contrast, Grothe’s four stages are based on the frequency of regional amyloid positivity across cognitively unimpaired individuals. The regional amyloid deposition in Grothe’s staging begins in inferior temporal and anterior cingulate areas, and progresses towards the remaining associative neocortex, followed by primary sensory-motor areas and the medial temporal lobe. The last stage of deposition is represented by the striatum. This is in line with Thal’s suggestion that amyloid deposition in the striatum is a marker of an advanced stage. Mattsson’s three stages are categorised into early, intermediate and late. The early stage of amyloid deposition includes the precuneus, posterior cingulate, as well as medial and lateral orbitofrontal cortices. Similarly to Grothe’s second stage, Mattsson’s intermediate stage includes associative cortices. Interestingly, this stage also comprises the cuneus, which is usually regarded as a late amyloid-accumulating area. In contrast to Mattsson, Grothe’s staging did not include occipital regions into the same stage as the associative neocortex. Mattson’s late amyloid stage includes the rest of the occipital lobe, including the lingual gyrus, but also the pericalcarine, and central cortices. Despite the methodological diversity, there is a clear regional overlap between all three methods, largely within default mode network regions including anterior cingulate, orbital-, middle-, superior-frontal gyrus, posterior cingulate, and insula.

# Inclusion Criteria for the Cognitively Unimpaired Elderly Cohort

The following inclusion criteria were applied: age of 60 years and older, a score of delayed recall above − 1.5 SD of demographically adjusted normative data on the Consortium to Establish a Registry for Alzheimer’s Disease (CERAD) 10-word list, a score of 23 or higher on the Telephone Interview for Cognitive Status-modified, a score of less than 11 on the 15-item Geriatric Depression Scale, as well as a Clinical Dementia Rating score of 0.

# Exclusion Criteria for the Cognitively Unimpaired Elderly Cohort

Individuals with the following medical conditions were excluded: diagnosis of mild cognitive impairment (MCI), probable Alzheimer’s disease or other neurodegenerative disorders, including Huntington disease, cortical basal degeneration, multiple system atrophy, Creutzfeldt-Jakob disease, primary progressive aphasia or Parkinson’s disease, stroke leading to physical disability, epilepsy with current use of antiepileptic drugs, brain infection (e.g., herpes simplex encephalitis), brain tumour, severe head injury or trauma with loss of consciousness for longer than 5 min, cancer with terminal life expectancy, untreated vitamin B12 deficiency, diabetes mellitus, thyroid disease, schizophrenia, bipolar disorders, as well as recurrent psychotic disorders. Other exclusion criteria included a history of recreational drug use, weekly alcohol consumption of more than 35 units per week (1 unit would equal to 10 ml or 8 g of pure alcohol), use of high-dose benzodiazepine, lithium carbonate, antipsychotics, high-dose antidepressants, or medication for Parkinson’s disease. Excluded were also individuals who could not come to the hospital due to physical morbidity or illness, as well as individuals who had a contraindication for MRI (e.g., metal implants, pacemaker, etc.).

# Blood plasma processing

Blood samples were collected and frozen on the same day as the PET scan and subsequently transported to Gothenburg. Plasma p-tau181 concentration was measured using the Simoa HD-X (Quanterix, Billerica, MA, USA) at the University of Gothenburg’s (Sweden) neurochemistry lab. For p-tau181, the AT270 mouse monoclonal antibody, that is specific to threonine-181 phosphorylation site, was coupled with magnetic beds, for capture. The detector was anti-tau mouse monoclonal antibody Tau12 (BioLegend, San Diego, CA, USA), which binds to the human tau protein at the N-terminal epitope 6-QEFEVMEDHAGT18. The detection antibody was conjugated to biotin following the manufacturer’s recommendations, and the calibrator was full-length recombinant tau-441 phosphorylated in vitro by glycogen synthase kinase.

# Centiloid processing

All ^18^F-flutemetamol PET SUVR data in the main paper were converted to the centiloid scale using a non-standard centiloid pipeline, Klunk et al’s^4^ instructions were followed to achieve this.

# Standard centiloid pipeline

The standard processing pipeline was validated first using the reference data. The ^11^C-PIB images from 34 young controls (YC) and 45 AD patients were downloaded from the GAAIN website ([www.gaain.org/centiloid-project](http://www.gaain.org/centiloid-project)). These images were processed with SPM8 according to the protocol. Centiloid cortex SUVR with cerebellar GM reference was calculated for both YC and AD groups. The YC mean SUVR = 1.17 ± 0.06 and the AD mean SUVR = 2.47 ± 0.28, both are within the 2% of Klunk et al’s published values. Cortical SUVRs were then converted to centiloids using Eq. 2.2.1 from ^1^. The final equation (Eq. S1) was CL = 100*(PIB_SUVR_ - 1.165) / (2.472 - 1.165). When calculated centiloid values were compared to the published ones all criteria were met (R^2^ = 0.99, slope = 0.998 and intercept = 0.138).

# Non-standard centiloid pipeline

A non-standard centiloid pipeline was established for the current study. The non-standard pipeline performed all ^11^C-PIB image sampling in native image space (defined by each person’s T1 MR image). To do this, centiloid cortical masks were inverse warped into each participant’s native image space using the _seg_inv_sn.mat file from SPM8’s segmentation. The inverse warped centiloid cortices were then multiplied with corresponding binary GM masks (GM density >50%). The bilateral GM of the cerebellum, as defined by the Hammers atlas ^5^ in native image space, was used as the reference region. Mean activity concentration (kBq/ml) was measured from coregistered ^11^C-PIB images for each individual’s centiloid GM mask and bilateral cerebellar GM to generate SUVRs. The non-standard SUVRs (mean YC SUVR = 1.16 ± 0.06 and mean AD SUVR = 2.39 ± 0.25) were converted to centiloid and correlated with the standard published centiloid values (Supplementary Fig. 2); all criteria were met for establishing a non-standard centiloid pipeline (R^2^ = 0.990, slope = 0.994 and intercept = 0.319).

## Supplementary Fig. 1 Published centiloid values vs locally generated values

## Supplementary Fig. 2 Published centiloid values vs non-standard local values

Following the validation of the non-standard pipeline and since the current work used ^18^F-flutemetamol the non-standard method needed to be calibrated against the ^11^C-PIB gold-standard. The 90-110min ^18^F-flutemetamol and 50-70 min ^11^C-PIB data acquired from the same individuals were downloaded from the GAAIN website ^6^. The two groups of images were processed according to the non-standard pipeline outlined above and SUVRs were calculated for both tracers. Linear regression of ^18^F-flutemetamol vs. ^11^C-PIB was performed. The resulting conversion from ^18^F-flutemetamol to ^11^C-PIB equivalent was (Eq. S2): PIB_eqSUVR_ = FLUT_SUVR_ – 0.2801/0.8232 (R^2^ = 0.937, slope = 0.823 and intercept = 0.280).

To calculate centiloid values ^18^F-flutemetamol cortical SUVR values were transformed to ^11^C-PIB equivalent SUVRs using Eq. S2 and then to centiloid using Eq. S1 (CL = 100*(PIB_eqSUVR_ - 1.161) / (2.388 - 1.161)).

## Supplementary Fig. 3 ^18^F-flutemetamol to 11C-PIB conversion with non-standard pipeline

# Supplementary Table 1 Cohort characteristics based on middle-, oldest-old and AT classification
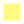


| Measure | Middle-old  (*n* = 38) | Oldest-old  (*n* = 37) | A-T-  (*n* = 27) | A+T-  (*n* = 24) | A-T+  (*n* = 7) | A+T+  (*n* = 15) |
| --- | --- | --- | --- | --- | --- | --- |
| Age (years) | 81.3 ± 1.52 | 88.5 ± 2.84**^a^** | 84.9 ± 4.09 | 83.2 ± 3.63 | 86.7 ± 4.23 | 86.1 ± 5.15 |
| Sex (F/M) | 29/9 | 29/8 | 21/6 | 22/2 | 3/4 | 10/5 |
| Education (years) | 14.4 ± 2.93 | 14 ± 3.05 | 15.0 ± 3.29 | 13.3 ± 2.62 | 15.5 ± 3.18 | 13.6 ± 2.47 |
| MMSE (/30) | 29 ± 1.06 | 29 ± 1.37 | 29.0 ± 0.94 | 28.6 ± 1.25 | 29.0 ± 1.00 | 28.5 ± 1.73 |
| ACE-R (/100) | 94 ± 4.13 | 92 ± 5.51 | 93.2 ± 3.76 | 93.2 ± 4.65 | 94.6 ± 4.35 | 91.7 ± 5.73 |
| ACE-Memory (/26) | 23.4 ± 2.54 | 23 ± 3.11 | 23.2 ± 2.69 | 24.0 ± 2.18 | 23.9 ± 1.86 | 22.1 ± 3.72 |
| CERAD Delayed (/10) | 8 ± 1.4 | 8 ± 1.68 | 7.93 ± 1.41 | 8.79 ± 1.06 | 7.29 ± 2.43 | 7.93 ± 1.75 |
| RAVLT Delayed (/15) | 11 ± 3.14 | 10 ± 3.5 | 10.3 ± 3.43 | 11.8 ± 2.87 | 9.57 ± 3.55 | 9.73 ± 3.58 |
| RCFT Delayed (/36) | 16.6 ± 5.78 | 13.2 ± 6.16**^a^** | 15.5± 4.66 | 15.3 ± 7.37 | 14.0 ± 6.96 | 14.1 ± 6.67 |
| Centiloid | 60.3 ± 57.2 | 38.0 ± 45.5 | 8.43 ± 11.9**^b,c^** | 74.2 ± 46.4 | 6.99 ± 12.4**^b,c^** | 105.2 ± 46.4 |
| p-tau181 (pg/mL; n = 73) | 15.4 ± 4.20 | 17.3 ± 6.79 | 12.6 ± 3.03**^c,d^** | 13.8 ± 2.36**^c,d^** | 22.6 ± 3.70 | 23.9 ± 3.58 |
|  | n (%) | n (%) | n (%) | n (%) | n (%) | n (%) |
| APOE E2/E4 | 3 (7.89) | - | - | - | 1 (1.37) | 2 (2.74) |
| APOE E3/E4 | 9 (23.7) | 5 (13.5) | 2 (2.74) | 7 (9.59) | 1 (1.37) | 4 (5.48) |
| APOE E4/E4 | 1 (2.63) | - | - | 1 (1.37) | - | - |

Values are mean ± standard deviation, or number of participants in a subset of the sample (*n*); MMSE, Mini-Mental State Examination; ACE-R, Addenbrooke’s Cognitive Examination-Revised; CERAD, Consortium to Establish a Registry for Alzheimer’s Disease; RAVLT, Rey Auditory Learning Test; RCFT Rey Complex Figure Test; CL, centiloid; APOE, apolipoprotein E.

**^a^** Significant difference (p < .05) between middle- and oldest-old individuals according to the independent-samples t-test.

**^b^** Significant pairwise difference (p < .005) to A+T- group according to Kruskal-Wallis (adjusted for multiple comparisons).

**^c^** Significant pairwise difference (p < .001) to A+T+ group according to Kruskal-Wallis (adjusted for multiple comparisons).

**^d^** Significant pairwise difference (p < .005) to A-T+ group according to Kruskal-Wallis (adjusted for multiple comparisons).

# Supplementary Table 2 Regional Mean SUVs and Principal Components Loadings for PCs1-3

| **Brain region** | **Mean SUV** | **PC1** | **PC2** | **PC3** |
| --- | --- | --- | --- | --- |
| Pons | 1.70 | 0.12 | 0.90 | 0.12 |
| Medulla | 1.34 | 0.09 | 0.82 | 0.01 |
| Midbrain | 1.27 | 0.11 | 0.90 | 0.14 |
| Presubgenual frontal cortex, r | 1.26 | 0.91 | 0.24 | 0.25 |
| Subgenual frontal cortex, r | 1.25 | 0.84 | 0.35 | 0.24 |
| Cingulate gyrus, anterior part, r | 1.23 | 0.91 | 0.23 | 0.32 |
| Cingulate gyrus, posterior part, r | 1.21 | 0.86 | 0.21 | 0.42 |
| Cingulate gyrus, posterior part, l | 1.18 | 0.87 | 0.24 | 0.39 |
| Putamen, r | 1.16 | 0.78 | 0.41 | 0.33 |
| Cingulate gyrus, anterior part, l | 1.15 | 0.91 | 0.21 | 0.32 |
| Inferior frontal gyrus, l | 1.15 | 0.88 | 0.29 | 0.34 |
| Insula, l | 1.15 | 0.79 | 0.41 | 0.40 |
| Putamen, l | 1.14 | 0.79 | 0.43 | 0.30 |
| Subgenual frontal cortex, l | 1.14 | 0.87 | 0.30 | 0.24 |
| Basal ganglia, r | 1.13 | 0.83 | 0.39 | 0.22 |
| Presubgenual frontal cortex, l | 1.13 | 0.92 | 0.23 | 0.23 |
| Inferior frontal gyrus, r | 1.13 | 0.85 | 0.29 | 0.38 |
| OFC, l | 1.13 | 0.89 | 0.27 | 0.34 |
| Middle frontal gyrus, r | 1.12 | 0.88 | 0.22 | 0.38 |
| Middle frontal gurus, l | 1.12 | 0.90 | 0.23 | 0.34 |
| OFC, r | 1.12 | 0.89 | 0.26 | 0.33 |
| Superior parietal gyrus, l | 1.11 | 0.81 | 0.27 | 0.47 |
| Inferiolateral remainder of parietal lobe, r | 1.10 | 0.79 | 0.25 | 0.51 |
| Insula, r | 1.09 | 0.81 | 0.40 | 0.40 |
| Superior parietal gyrus, r | 1.09 | 0.77 | 0.23 | 0.53 |
| Middle and inferior temporal gyrus, l | 1.09 | 0.80 | 0.34 | 0.456 |
| Inferiolateral remainder of parietal lobe, l | 1.09 | 0.82 | 0.27 | 0.45 |
| Superior frontal gyrus, r | 1.09 | 0.89 | 0.24 | 0.35 |
| Posterior temporal lobe, l | 1.07 | 0.76 | 0.35 | 0.50 |
| Posterior temporal lobe, r | 1.07 | 0.75 | 0.30 | 0.57 |
| Precentral gyrus, l | 1.06 | 0.70 | 0.44 | 0.50 |
| Superior temporal gyrus, posterior part, l | 1.06 | 0.78 | 0.39 | 0.43 |
| Postcentral gyrus, l | 1.06 | 0.71 | 0.41 | 0.52 |
| Superior frontal gyrus, l | 1.06 | 0.90 | 0.24 | 0.32 |
| Basal ganglia, l | 1.06 | 0.83 | 0.40 | 0.19 |
| Postcentral gyrus, r | 1.05 | 0.69 | 0.39 | 0.56 |
| Precentral gyrus, r | 1.05 | 0.68 | 0.43 | 0.53 |
| Superior temporal gyrus, posterior part, r | 1.04 | 0.78 | 0.34 | 0.46 |
| Lateral remainder of occipital lobe, r | 1.04 | 0.51 | 0.39 | 0.72 |
| Middle and inferior temporal gyrus, r | 1.04 | 0.79 | 0.32 | 0.47 |
| Lateral remainder of occipital lobe, l | 1.03 | 0.58 | 0.46 | 0.62 |
| Fusiform gyrus, r | 1.02 | 0.63 | 0.41 | 0.61 |
| Fusiform gyrus, l | 0.99 | 0.67 | 0.47 | 0.51 |
| Thalamus, l | 0.96 | 0.53 | 0.77 | 0.09 |
| Caudate nucleus, r | 0.95 | 0.91 | 0.26 | 0.16 |
| Thalamus, r | 0.93 | 0.57 | 0.70 | 0.12 |
| Cuneus, r | 0.93 | 0.50 | 0.43 | 0.69 |
| Cuneus, l | 0.92 | 0.58 | 0.46 | 0.59 |
| Amygdala, r | 0.91 | 0.45 | 0.70 | 0.37 |
| Anterior temporal lobe, lateral part, l | 0.91 | 0.78 | 0.33 | 0.49 |
| Parahippocampal and ambient gyri, r | 0.91 | 0.48 | 0.71 | 0.42 |
| Hippocampus, r | 0.91 | 0.37 | 0.75 | 0.34 |
| Lingual gyrus, r | 0.90 | 0.47 | 0.45 | 0.71 |
| Lingual gyrus, l | 0.90 | 0.55 | 0.49 | 0.58 |
| Anterior temporal lobe, lateral part, r | 0.90 | 0.78 | 0.29 | 0.45 |
| Hippocampus, l | 0.89 | 0.43 | 0.73 | 0.32 |
| Caudate nucleus, l | 0.88 | 0.89 | 0.29 | 0.17 |
| Parahippocampal and ambient gyri, l | 0.88 | 0.50 | 0.68 | 0.38 |
| Superior temporal gyrus, anterior part, r | 0.87 | 0.82 | 0.28 | 0.39 |
| Anterior temporal lobe, medial part, r | 0.86 | 0.65 | 0.47 | 0.52 |
| Amygdala, l | 0.86 | 0.48 | 0.70 | 0.38 |
| Superior temporal gyrus, anterior part, l | 0.85 | 0.79 | 0.32 | 0.45 |
| Subcallosal area, r | 0.85 | 0.84 | 0.32 | 0.16 |
| Anterior temporal lobe, medial part, l | 0.83 | 0.67 | 0.49 | 0.48 |
| Subcallosal area, l | 0.79 | 0.85 | 0.21 | 0.12 |
| Cerebellum, l | 0.68 | 0.11 | 0.88 | 0.26 |
| Cerebellum, r | 0.66 | 0.16 | 0.84 | 0.29 |

The Hammers’ atlas regions are ordered from highest to lowest mean SUV. Darker colours represent loadings above .7, lighter colours represent loadings above .5. Abbreviations: r = right; l = left; Basal ganglia, composite region comprising nucleus accumbens, substantia nigra, pallidum; OFC, composite region comprising straight gyrus, anterior orbital gyrus, medial orbital gyrus, lateral orbital gyrus, posterior orbital gyrus.


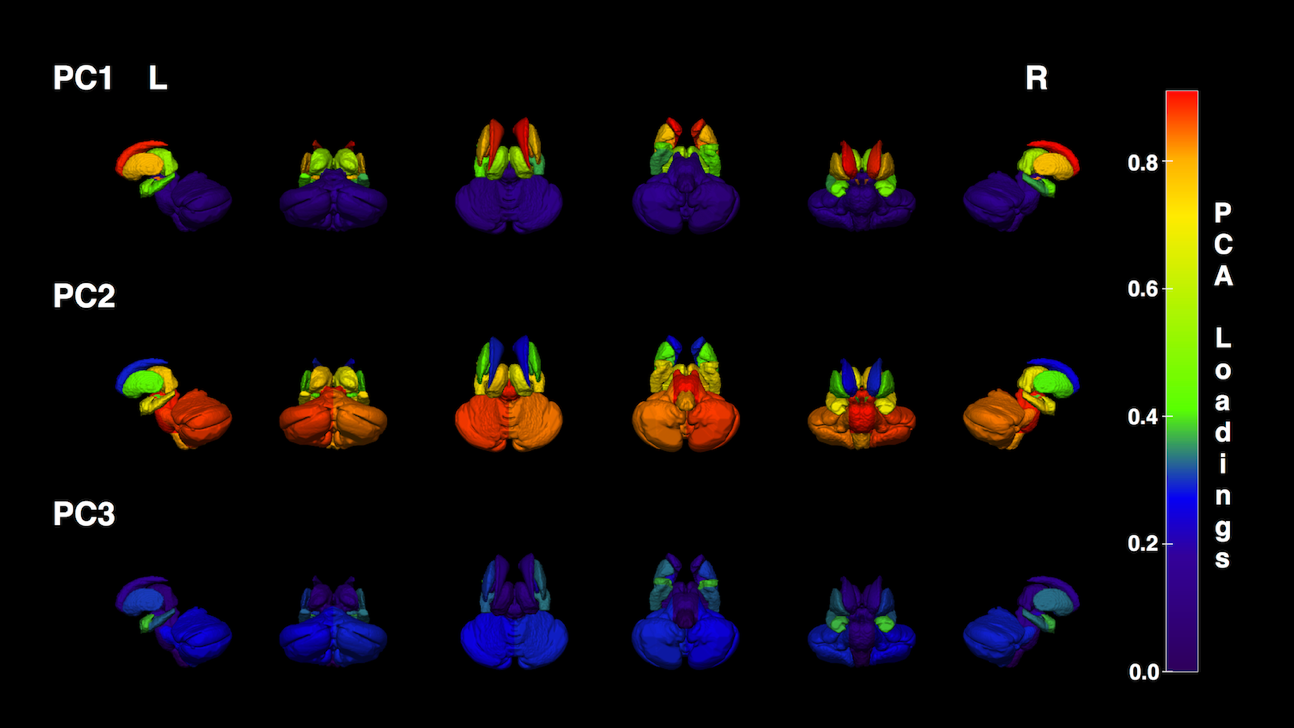


# Supplementary Fig. 4 Representation of regional loadings from the first three PCs for subcortical regions not visible in Fig. 1

Each Hammer’s atlas region was scaled to its loading score from the PCA. Note, that caudate is clearly the highest subcortical region loaded onto PC1 with values in the same range as frontal neocortex. Brainstem and cerebellum are highly loaded onto PC2 and all subcortical regions have low loadings for PC3. Colour bar represents PC loadings from highest (red) to lowest (violet). Abbreviations: PC, principal component; L, left; R, right.


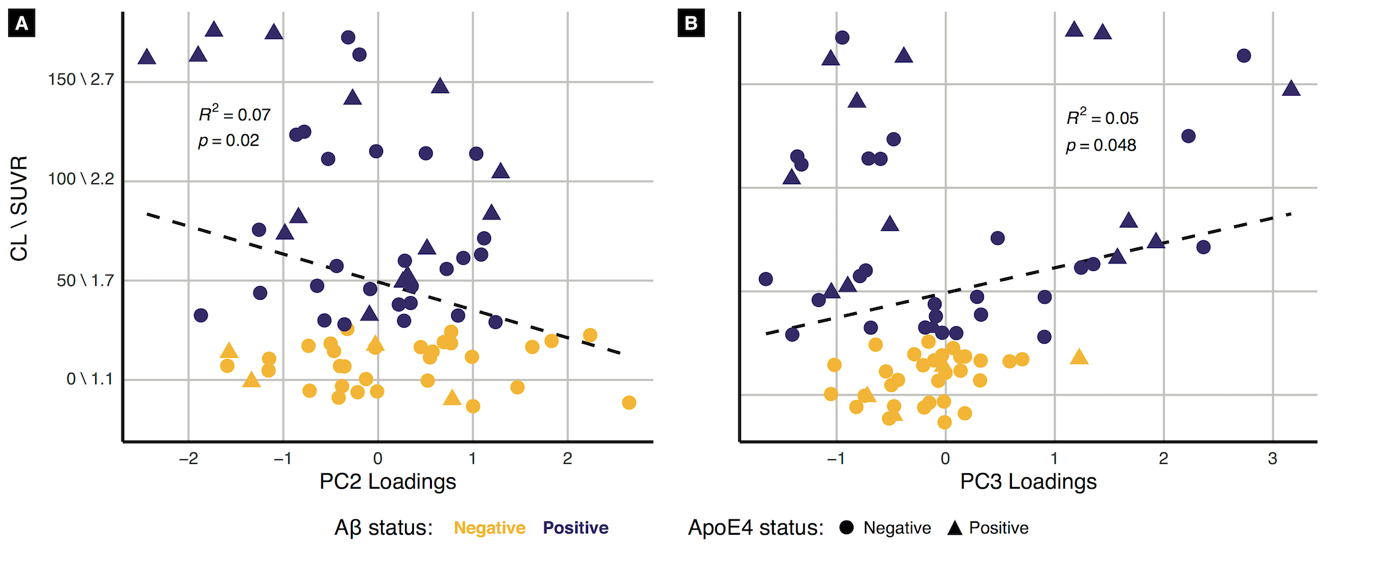


# Supplementary Fig. 5 Scatter plots displaying the significant correlation between global CL \ SUVRs, PC2 (A) and PC3 (B)

In both A & B triangles represent APOE4 carriers and circles APOE4 non-carriers. One participant’s APOE status was unknown and is represented by a square in A & B. Binary Aβ status is depicted in colour (Aβ- = yellow; Aβ+ = violet).


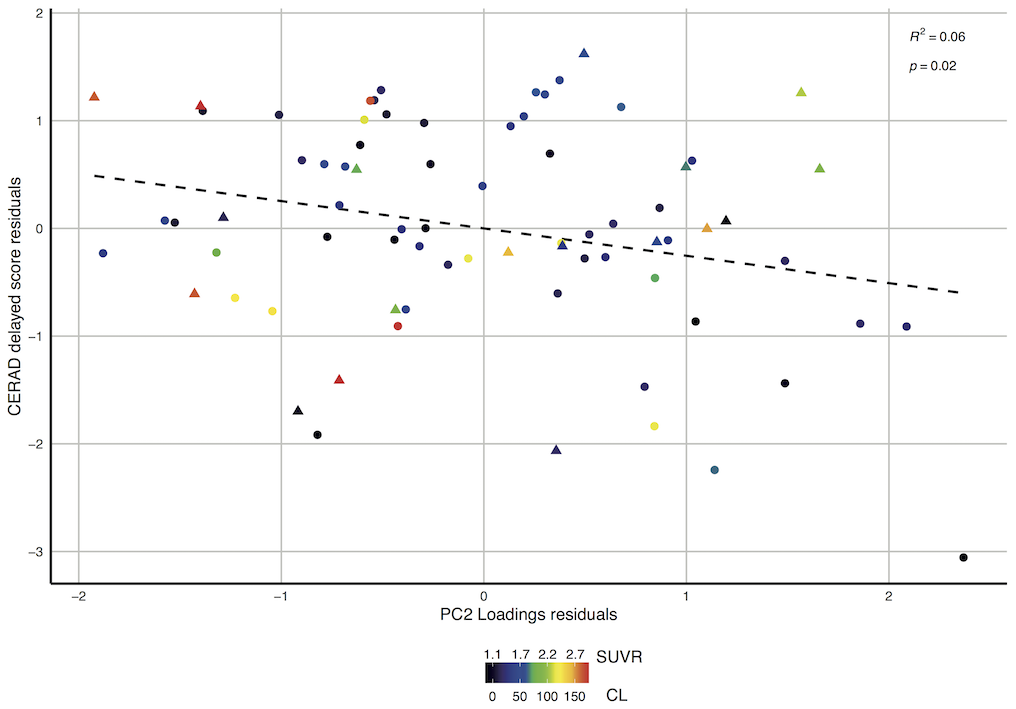


# Supplementary Fig. 6 Scatter plot showing the significant partial correlation (adjusted for age, years of education and APOE4) between delayed CERAD and PC2

Triangles represent APOE4 carriers and circles APOE4 non-carriers, points are coloured by continuous CL \ SUVRs, represented by the colour bar.


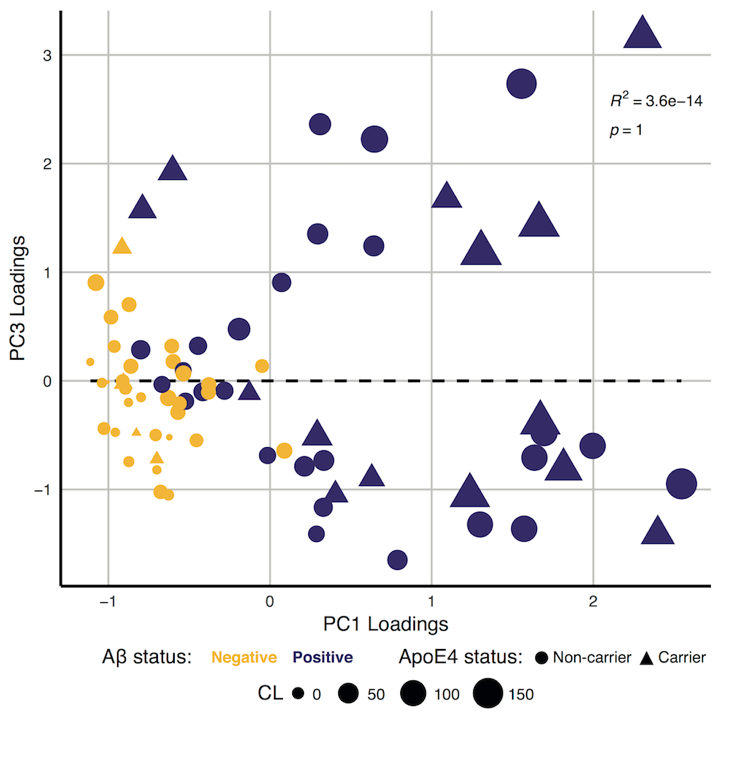


# Supplementary Fig. 7 Scatter plot showing the relationship between PC1 and PC3

Triangles represent APOE4 carriers and circles APOE4 non-carriers. Binary Aβ status is depicted in colour (Aβ- = yellow; Aβ+ = violet). The size of each point is proportional to the CL value.

# References

1. Thal DR, Beach TG, Zanette M, Lilja J, Heurling K, Chakrabarty A et al. Estimation of amyloid distribution by [18 F] flutemetamol PET predicts the neuropathological phase of amyloid β-protein deposition. Acta neuropathologica 2018; 136(4): 557-567.

2. Grothe MJ, Barthel H, Sepulcre J, Dyrba M, Sabri O, Teipel SJ et al. In vivo staging of regional amyloid deposition. Neurology 2017; 89(20): 2031-2038.

3. Mattsson N, Palmqvist S, Stomrud E, Vogel J, Hansson O. Staging β-amyloid pathology with amyloid positron emission tomography. JAMA neurology 2019; 76(11): 1319-1329.

4. Klunk WE, Koeppe RA, Price JC, Benzinger TL, Devous Sr MD, Jagust WJ et al. The Centiloid Project: standardizing quantitative amyloid plaque estimation by PET. Alzheimer's & dementia 2015; 11(1): 1-15. e14.

5. Hammers A, Chen CH, Lemieux L, Allom R, Vossos S, Free SL et al. Statistical neuroanatomy of the human inferior frontal gyrus and probabilistic atlas in a standard stereotaxic space. Hum Brain Mapp 2007; 28(1): 34-48.

6. Battle MR, Pillay LC, Lowe VJ, Knopman D, Kemp B, Rowe CC et al. Centiloid scaling for quantification of brain amyloid with [(18)F]flutemetamol using multiple processing methods. EJNMMI Res 2018; 8(1): 107.
